# P-1874. Measuring the Clinical Impact of a Novel Infectious Disease Step-Down Service

**DOI:** 10.1093/ofid/ofaf695.2043

**Published:** 2026-01-11

**Authors:** Makenzie Starlin, Richard Starlin, Trevor C Van Schooneveld, Bryan T Alexander, Molly M Miller, Kaeli Samson

**Affiliations:** University of Nebraska Medical Center, Omaha, NE; University of Nebraska Medical Center, Omaha, NE; University of Nebraska Medical Center, Omaha, NE; Nebraska Medicine, Omaha, Nebraska; Nebraska Medicine, Omaha, Nebraska; University of Nebraska Medical Center, Omaha, NE

## Abstract

**Background:**

Non-adherence to ID recommendations occurs frequently following ID sign-off and can result in adverse patient events. An ID Step-Down Service (SDS) was created to follow patients daily who continued to receive antimicrobial therapy in the hospital after primary ID team sign-off. Patients are followed to therapy completion, discharge or transition to the primary ID service. Since 2019, the Outpatient Parenteral Antimicrobial Therapy (OPAT) team has also regularly reviewed inpatients on intravenous antibiotics after ID sign-off.

**Table 1:**
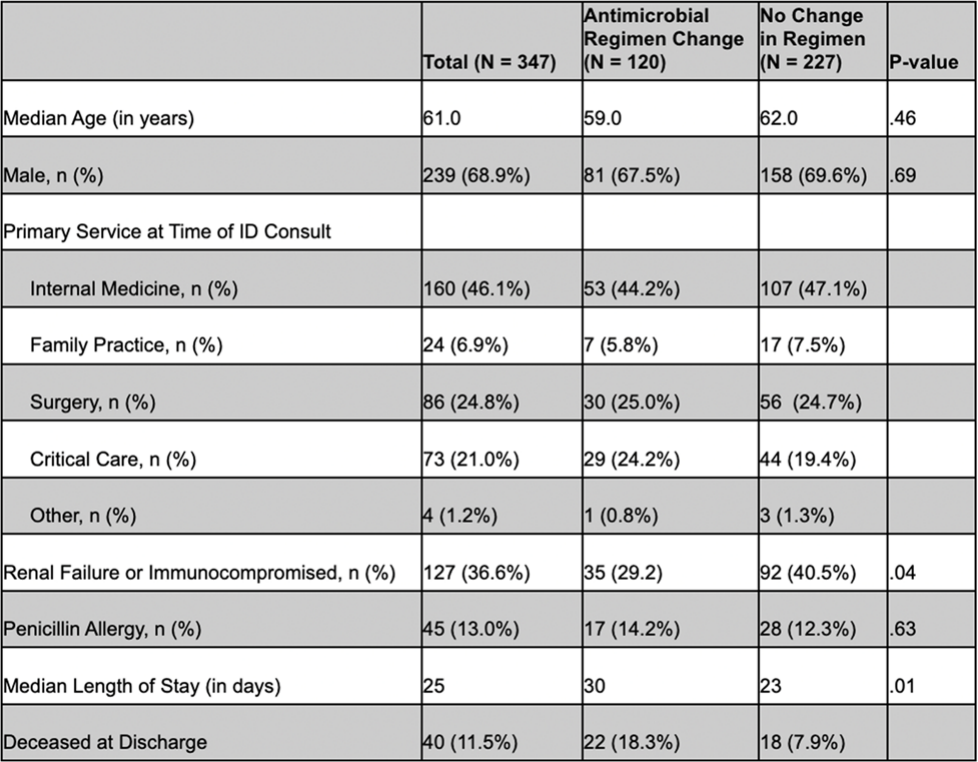
Patient Demographics and Comparison of Patients With and Without Antimicrobial Regimen Change After Primary ID Service Sign Off

**Table 2:**
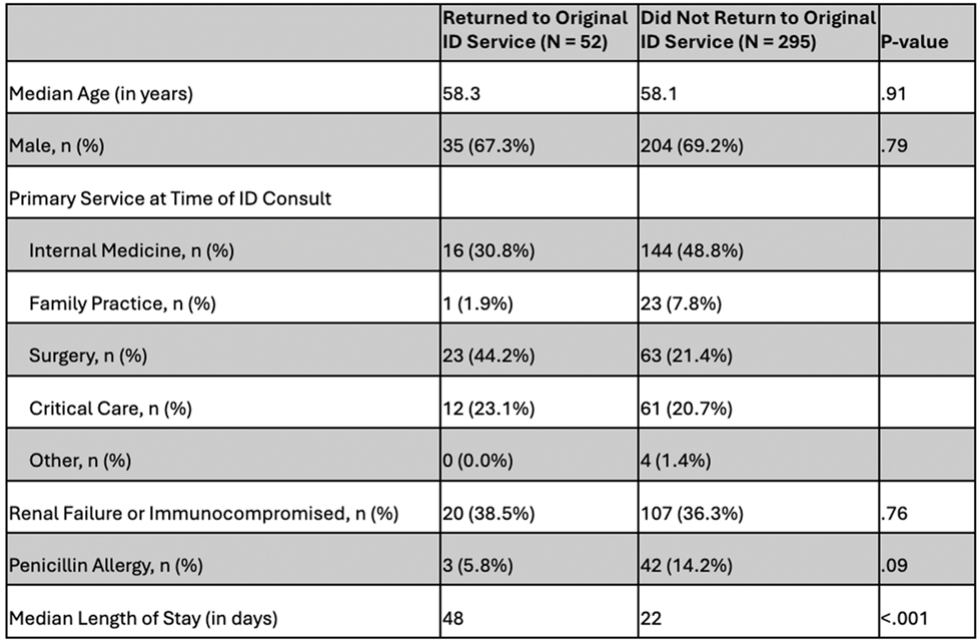
Patients With and Without Return to Original ID Consult Service

**Methods:**

We performed a retrospective review of 347 patients followed by the SDS from 7/1/2022 to 12/31/2023 identifying unplanned antimicrobial changes, determining who directed the change, and identifying return to the primary ID service. We compared those with a regimen change or return to the primary ID service to those without. We reviewed 945 patients followed by OPAT pre-SDS (1/1/21-6/30/22) measuring frequency of antimicrobial interventions and re-consultation of the primary ID service.

**Results:**

Unplanned antimicrobial changes occurred in 34.6% (120/347) of patients followed by SDS (Table 1). The SDS directed the change 69.2% (83/120) of the time. Unplanned medication changes were associated with increased length of stay and a greater number of deceased patients at discharge. Re-consultation of the primary ID service occurred in 15.0% (52/347) and was associated with a significantly increased length of hospital stay (p< .001). Of the 945 patients followed by the OPAT service, 18.9% (179/945) had an antimicrobial intervention to adjust therapy, and the primary ID service was re-consulted 4.7% (44/945) of the time.

**Conclusion:**

Unplanned regimen changes are common after primary ID service sign off, and the SDS plays an important role in directing changes and re-engaging the primary ID team. ID directed regimen changes and re-engagement of the primary ID team were increased compared to monitoring by the OPAT service alone. These findings support the role of the SDS to clinically follow patients receiving antimicrobial therapy in the hospital after sign-off by the primary ID service.

**Disclosures:**

Trevor C. Van Schooneveld, MD, FSHEA, FIDSA, BioMerieux: Advisor/Consultant|BioMerieux: Grant/Research Support Bryan T. Alexander, PharmD, BCIDP, AAHIVP, Astellas Pharma: Advisor/Consultant|Merck: Grant/Research Support

